# Substitute or coexistence? Mediastinoscopy-assisted versus thoracoscope-assisted esophagectomy in esophageal cancer: a meta-analysis of perioperative outcomes and long-term survival

**DOI:** 10.1097/JS9.0000000000001777

**Published:** 2024-06-13

**Authors:** Pinhao Fang, Jianfeng Zhou, Yixin Liu, Zhiwen Liang, Yushang Yang, Siyuan Luan, Xin Xiao, Xiaokun Li, Hanlu Zhang, Qixin Shang, Longqi Chen, Xiaoxi Zeng, Yong Yuan

**Affiliations:** a Department of Thoracic Surgery, Med+X Center for Informatics, West China Hospital, Sichuan University; b West China Biomedical Big Data Center, Med+X Center for Informatics, West China Hospital, Sichuan University, Chengdu, China

**Keywords:** esophageal cancer, mediastinoscopy-assisted esophagectomy, meta-analysis, perioperative outcomes, survival, thoracoscope-assisted esophagectomy

## Abstract

**Background:**

Currently, mediastinoscopy-assisted esophagectomy (MAE) and thoracoscope-assisted esophagectomy (TAE) represent two prevalent forms of minimally invasive esophagectomy extensively employed in the management of esophageal cancer (EC). The aim of this meta-analysis is to assess and compare these two surgical approaches concerning perioperative outcomes and long-term survival, offering valuable insights for refining surgical strategies and enhancing patient outcomes in this field.

**Methods:**

Adhering to PRISMA guidelines, the authors systematically searched PubMed, Web of Science, Cochrane Library, Embase, and CNKI databases until 1 March 2024, for studies comparing MAE and TAE. Outcomes of interest included perioperative outcomes (intraoperative outcomes, postoperative recovery, postoperative complications) and survival rates. Statistical analyses were performed using RevMan 5.4, with heterogeneity dictating the use of fixed or random-effects models.

**Results:**

A total of 21 relevant studies were finally included. MAE was associated with significantly shorter operation times [mean difference (MD)=−59.58 min, 95% CI: −82.90 to −36.26] and less intraoperative blood loss (MD=−68.34 ml, 95% CI: −130.45 to −6.23). However, MAE resulted in fewer lymph nodes being dissected (MD=−3.50, 95% CI: −6.23 to −0.78). Postoperative recovery was enhanced following MAE, as evidenced by reduced hospital stays and tube times. MAE significantly reduced pulmonary complications [odds ratio (OR)=0.59, 95% CI: 0.44, 0.81] but increased the incidence of recurrent laryngeal nerve injury (OR=1.84, 95% CI: 1.30, 2.60). No significant differences were observed in anastomotic leakage, chylothorax, cardiac complications, wound infections, and gastric retention between MAE and TAE. The long-term survival outcomes showed no statistical difference [hazard ratio (HR)=1.05, 95% CI: 0.71, 1.54].

**Conclusions:**

MAE offers advantages in reducing operation time, blood loss, and specific postoperative complications, particularly pulmonary complications, with a shorter recovery period compared to TAE. However, it poses a higher risk of recurrent laryngeal nerve injury and results in fewer lymph nodes being dissected. No difference in long-term survival was observed, indicating that both techniques have distinct benefits and limitations. These findings underscore the need for personalized surgical approaches in EC treatment, considering individual patient characteristics and tumor specifics.

## Introduction

Highlights

- This meta-analysis offers a comprehensive view on mediastinoscopy-assisted esophagectomy (MAE) versus thoracoscope-assisted esophagectomy (TAE).
- The study reveals MAE’s significant advantages over TAE in reducing operation time, intraoperative blood loss, and specific postoperative complications, particularly pulmonary complications, and MAE is associated with a shorter recovery period, including reduced hospital stays and tube times.
- Despite its benefits, MAE is linked to fewer lymph nodes being dissected and an increased incidence of recurrent laryngeal nerve injury.
- Our meta-analysis stands out as the first to comprehensively integrate and analyze long-term survival outcomes indicating no significant difference in long-term survival rates between patients underwent MAE and TAE.

Esophageal cancer (EC) stands as the ninth most common cancer worldwide. According to pertinent reports, there were 604 000 newly diagnosed cases and 540 000 deaths attributed to EC in 2020^1^. Due to the insidious nature of its symptoms, EC often remains undetected until it has reached an advanced stage. Consequently, patients diagnosed with EC face a grim prognosis, with the five-year survival rate persisting below 30%^2^, which presents a significant threat to individuals’ health. This stark reality underscores the urgent need for effective treatment modalities, with surgical intervention playing a pivotal role in the management of localized EC^3^.

In recent decades, there has been notable advancement in minimally invasive esophagectomy. Thoracoscope-assisted esophagectomy (TAE) stands as a significant application of modern surgical techniques in the treatment of patients with EC and has gained widespread adoption in clinical practice. In contrast to traditional open transthoracic esophagectomy, TAE demonstrates the potential to substantially minimize surgical trauma^4,5^. Nonetheless, to create sufficient space for TAE, single lung ventilation and artificial pneumothorax are imperative, which could potentially exacerbate pulmonary trauma, particularly in patients with compromised cardiopulmonary function or elderly individuals. Additionally, unilateral pulmonary ventilation may contribute to postoperative pulmonary complications, while transthoracic approaches could inevitably result in damage to the chest wall^6^. In recent years, there has been a gradual adoption of mediastinoscopy-assisted esophagectomy (MAE) in the surgical management of EC, aimed at mitigating the potential damage associated with TAE. A pivotal milestone occurred in 1997 when Bumm *et al.* ^7^ pioneered the application of MAE in the resection of EC, thereby challenging the conventional surgical treatment paradigm for this cancer. In MAE, surgeons insert the mediastinoscopy into the upper and middle esophagus through the neck, thereby avoiding injury to the chest wall. With the assistance of mediastinoscopy, the thoracic segment of the esophagus is released through the posterior mediastinum without interrupting breathing and oxygenation. Consequently, the impact of unilateral lung ventilation on patients’ cardiopulmonary system is alleviated, providing surgical opportunities for EC patients with compromised cardiopulmonary function, particularly for those who may not tolerate reduced oxygenation. As a result, MAE has gradually emerged as an optional surgical approach of esophagectomy, especially for elderly patients^8^.

Nevertheless, MAE comes with its own set of challenges, including a narrow operating space and difficult local exposure, some surgeons have raised doubts regarding whether MAE can effectively replace traditional TAE^9^. Despite the growing body of literature on MAE and TAE, disparities in reported perioperative and survival outcomes necessitate a comprehensive evaluation to elucidate their comparative effectiveness. To address this, Sheng and colleagues conducted a meta-analysis comparing TAE and MAE^10^, their meta-analysis only included 8 studies with 733 patients, and the date of selected studies was up to 2021. Moreover, certain perioperative outcomes and postoperative complications of EC patients who underwent TAE and MAE have not been thoroughly investigated because of limited included studies. Notably, it completely omitted the examination of long-term survival rates of EC patients subjected to TAE and MAE. Therefore, leveraging updated research, we conducted this meta-analysis to comprehensively compare the perioperative and survival outcomes between MAE and TAE. We aim to offer the latest conclusions and clinical evidence to inform practice.

## Methods

This meta-analysis was conducted in accordance with the Preferred Reporting Items for Systematic Reviews and Meta-Analyses (PRISMA, Supplemental Digital Content 1, http://links.lww.com/JS9/C749, Supplemental Digital Content 2, http://links.lww.com/JS9/C750) guidelines and assessed the methodological quality using the Assessing the Methodological Quality of Systematic Reviews (AMSTAR, Supplemental Digital Content 3, http://links.lww.com/JS9/C751) guidelines^11,12^. Furthermore, this meta-analysis was registered in the PROSPERO database.

### Search design

PubMed, Web of Science, Cochrane Library, Embase, and CNKI were each independently searched. The search strategy was formulated using the following keywords: “esophageal cancer,” “mediastinoscopy-assisted esophagectomy,” and “thoracoscope-assisted esophagectomy.” Our literature search was finalized as of 1 March 2024, and encompassed pertinent research findings available up to this date. Reviews, expert opinions, case reports, letters, and conference literature were excluded from the search results.

### Study selection

Studies meeting the following criteria were included in the meta-analysis: (1) patients diagnosed with EC through pathological assessment; (2) studies categorizing EC patients into MAE and TAE groups; (3) studies comparing perioperative, or survival outcomes of patients who underwent MAE and TAE, including both prospective and retrospective studies; (4) language of the included studies was not restricted. Conversely, studies were excluded if they: (1) were duplicates; (2) involved animal experiments; and (3) did not report complete data or data that could not be extracted. Two researchers independently conducted database searches. The data on relevant outcomes were extracted and cross-checked by both researchers.

### Data extraction

Two scholars were responsible for extracting relevant data from the selected studies. In the event of conflicts, team discussions were conducted to resolve discrepancies. The retrieved data included: the author’s name, year of publication, study design, number of patients, patients’ age, cancer type, tumor location, tumor stage, and follow-up duration. Mean and standard deviation (SD) were employed as measures for comparing measurement data between MAE and TAE groups. Odds ratios (ORs) with their corresponding 95% CIs were used to evaluate enumeration data regarding postoperative complications. Hazard ratios (HRs) were extracted from the text of studies or obtained from Kaplan–Meier curves using Engauge Digitizer software.

### Quality assessment

The Newcastle–Ottawa Scale (NOS) was utilized to assess the quality of observational studies. Studies scoring higher than 6 on the NOS were regarded as high quality. Furthermore, the risk of bias in randomized controlled trials was evaluated in accordance with the standards outlined in the Cochrane Risk Bias Assessment Tool.

### Statistical analyses

Forest plots were employed to illustrate the effect size of the included studies in this meta-analysis. Depending on the heterogeneity of the analyzed data, different models were selected to pool the effect size of each included study. If the I^2^ values exceeded 50%, indicating substantial heterogeneity among the included studies, the random-effects model was employed for analysis. Conversely, if the I^2^ values were below 50%, indicating insignificant heterogeneity, the fixed-effects model was utilized. ORs with their corresponding 95% CIs were calculated from the categorical data.

Sensitivity analysis of the included studies was performed by systematically removing each study in turn and recalculating the pooled effect size. This was done to assess the impact of each study on the overall outcomes. Additionally, the *P* value and asymmetry of funnel plots were evaluated using Egger’s test to assess publication bias among the included studies. Statistical analyses were carried out using the RevMan 5.4 software package.

## Results

### Search results

**Figure 1:**
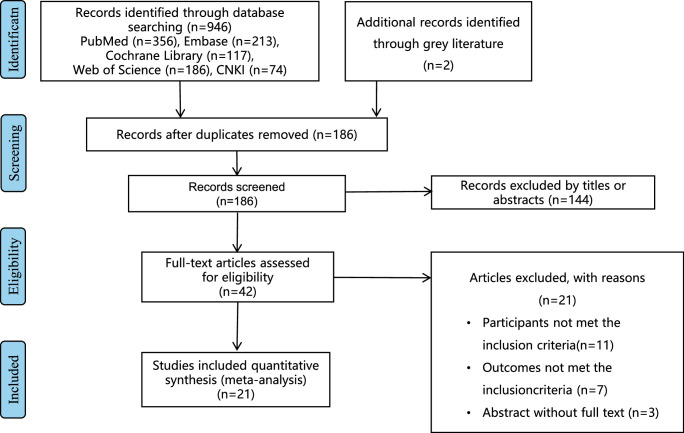
The flow diagram showing the process of study selection.

The analysis encompassed a comprehensive review of 21 studies, delineated into 3 randomized controlled trials (RCTs)^9,13,14^, 2 non-randomized controlled trials (nRCTs)^15,16^, and 16 retrospective studies^4,17–31^. The progression of literature screening is visually depicted in Figure 1, elucidating the inclusion process. Detailed characteristics of the incorporated studies are meticulously outlined in Table 1. A collective cohort of 1724 patients diagnosed with EC was amassed, with 838 underwent MAE and 886 underwent TAE. Notably, a predominant portion of the patient population hailed from East Asia. Further examination revealed that 15 studies were exclusively centered on patients subjected to surgical therapy, while 6 studies reported outcomes of patients who underwent surgical resection supplemented with additional adjuvant therapies.

**Table 1:** The characteristics of the included studies.

Name	Year	Country	Design	Cancer type	Treatment	TAE type	*N*		Male	Female	Tumor location						Stage	Age		NOS
											Upper		Middle		Lower					
							MAE	TAE			MAE	TAE	MAE	TAE	MAE	TAE		MAE	TAE	
Wang *et al*.^15^	2022	China	nRCT	ESCC	S	M	30	30	43	17	3	3	20	22	7	5	I–III	68.60±8.23	67.73±8.83	8
Wang *et al*.^17^	2018	China	RCS	ESCC	S + A	I	19	19	25	13	0	0	13	15	6	4	II–IV	63.1±7.1	63.2±4.8	7
Shi *et al*.^9^	2022	China	RCT	ESCC	S	TAE	100	100	128	72	14	15	66	68	20	17	I–III	66.3±6.7	66.3±6.1	^a^
Ken *et al*.^18^	2022	Japan	RCS	EC	N + S	M	34	38	61	11	8	6	13	26	13	6	0–IV	67 (46–77)	66.5 (51–81)	7
Li *et al*.^19^	2021	China	RCS	ESCC	S	TAE	28	48	61	15	/	/	/	/	/	/	0–III	66.71±8.10	63.69±6.03	8
Ma *et al*.^16^	2021	China	nRCT	ESCC	S	M	33	32	65	0	0	0	18	19	15	13	I–III	57.4±7.3	60.7±6.4	7
Feng *et al*.^20^	2012	China	RCS	ESCC	S	TAE	27	27	42	12	2	4	19	18	6	5	0–IV	58.6 (37–79)	61.1 (46–76)	7
Naohiko *et al*.^21^	2012	Japan	RCS	EC	N + S	TAE	17	37	43	11	1	3	11	27	5	7	I–II or more	66.3±12.9	65.3±8.9	7
Wang *et al*.^22^	2014	China	RCS	ESCC	S	TAE	109	58	92	75	/	/	/	/	/	/	I–II	62 (54–78)	62 (55–72)	7
Tsutomu *et al*.^23^	2016	Japan	RCS	EC	N + S	TAE	20	15	22	13	3	1	13	10	6	2	/	Mean 64	Mean 66	6
Liu *et al*.^24^	2020	China	RCS	ESCC	N + S	TAE	30	68	81	17	/	/	/	/	/	/	I–IV	58.03±8.79	56.97±8.88	8
Jin *et al*.^4^	2019	China	RCS	EC	S	TAE	19	30	44	5	10	19	7	9	2	2	I–III	62.50±8.46	59.74±7.92	6
Huang *et al*.^25^	2023	China	RCS	EC	S	M	38	38	56	20	2	3	22	21	14	14	I–III	67.7±7.8	67.8±9.6	6
Chen *et al*.^26^	2021	China	RCS	ESCC	S + A	TAE	51	51	84	17	6	5	32	30	13	16	I–III	65.5±7.3	64.1±7.0	7
Fang *et al*.^27^	2021	China	RCS	EC	S	TAE	43	59	88	14	5	6	23	29	15	24	I–IV	66.7±6.7	63.3±7.6	6
Wang *et al*.^28^	2023	China	RCS	ESCC	S	TAE	30	30	42	18	/	/	/	/	/	/	I–II	67.1±6.78	63±8.48	6
Tan *et al*.^29^	2003	China	RCS	ESCC	S	I	32	28	55	5	11	9	21	19	/	/	I–III	58.5±8.7	56.9±7.6	6
Xing *et al*.^30^	2013	China	RCS	EC	S	TAE	46	46	64	28	/	/	/	/	/	/	/	46-84	47-82	6
Yang *et al*.^13^	2021	China	RCT	EC	S	M	32	32	39	25	/	/	/	/	/	/	/	64.78±6.09	65.76±6.21	^a^
Xia *et al*.^14^	2021	China	RCT	EC	S	M	40	40	41	39	18	19	16	15	6	6	/	65.75±3.11	65.71±3.14	^a^
Fan *et al*.^31^	2021	China	RCS	EC	S	M	60	60	83	37	15	8	35	40	10	12	0–III	69.3±7.8	67.8±6.4	6

A, adjuvant therapy; EC, esophageal cancer; ESCC, esophageal squamous cell cancer; I, Ivor-Lewis; M, McKeown; MAE, mediastinoscopy-assisted esophagectomy; N, neoadjuvant therapy; NOS, Newcastle–Ottawa Quality Assessment Scale; nRCT, non-randomized controlled trial; RCS, retrospective cohort studies; RCT, randomized controlled trial; S, surgery; TAE, thoracoscope-assisted esophagectomy.

a The detailed results of Cochrane Collaboration’s Risk of Bias of two RCTs included in this meta-analysis were shown in Figure S1, Supplemental Digital Content 4, http://links.lww.com/JS9/C752.

### Quality assessment

The results of the NOS and Cochrane Collaboration’s Risk of Bias Tool were delineated in Table 1, stratified based on distinct study designs. Studies attaining an NOS score of greater than or equal to 6 were classified as high-quality investigations. Additionally, the RCTs underwent evaluation utilizing the Cochrane Risk Bias Assessment Tool, with a visual representation of the outcomes presented in Figure S1, Supplemental Digital Content 4, http://links.lww.com/JS9/C752.

### Intraoperative outcomes

**Figure 2:**
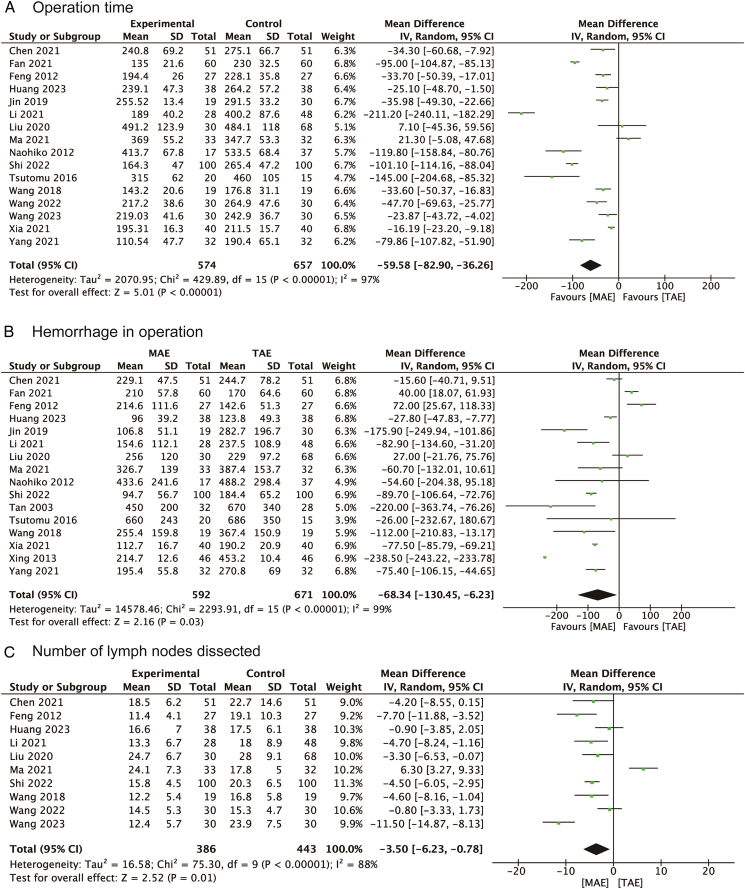
Forest plots of studies evaluating MDs of MAE versus TAE on intraoperative outcomes of esophageal cancers, stratified by (A) operation time; (B) hemorrhage in operation; (C) number of lymph nodes dissected. MAE, mediastinoscopy-assisted esophagectomy; TAE, thoracoscope-assisted esophagectomy.

The analysis demonstrated significant benefits of MAE over TAE in several key intraoperative outcomes. Specifically, 16 studies provided data on the total operation time for both MAE and TAE in patients with EC. Given that the heterogeneity among these studies was substantial, as indicated by an I^2^ value over 50%, a random-effects model was employed for the analysis. This analysis revealed that MAE was associated with a significantly shorter operation time than TAE, as evidenced by a mean difference (MD) of −59.58 min (95% CI: −82.90 to −36.26), as shown in Figure 2A. In terms of intraoperative blood loss, which was examined in 16 studies, the random-effects model was again applied due to significant heterogeneity (I^2^>50%). The results of this analysis highlighted that patients in the MAE group experienced significantly less blood loss compared to those in the TAE group, with an MD of −68.34 ml (95% CI: −130.45 to −6.23), as depicted in Figure 2B.

However, when considering the total number of lymph nodes dissected, an analysis of 10 studies showed that the count was significantly lower in the MAE group than in the TAE group. This was represented by an MD of −3.50 lymph nodes (95% CI: −6.23 to −0.78), according to the pooled data shown in Figure 2C. This sequence of results underscores the benefits of MAE in terms of reduced operation time and intraoperative blood loss, albeit with a decrease in the number of lymph nodes dissected when compared to TAE.

### Postoperative recovery

**Figure 3:**
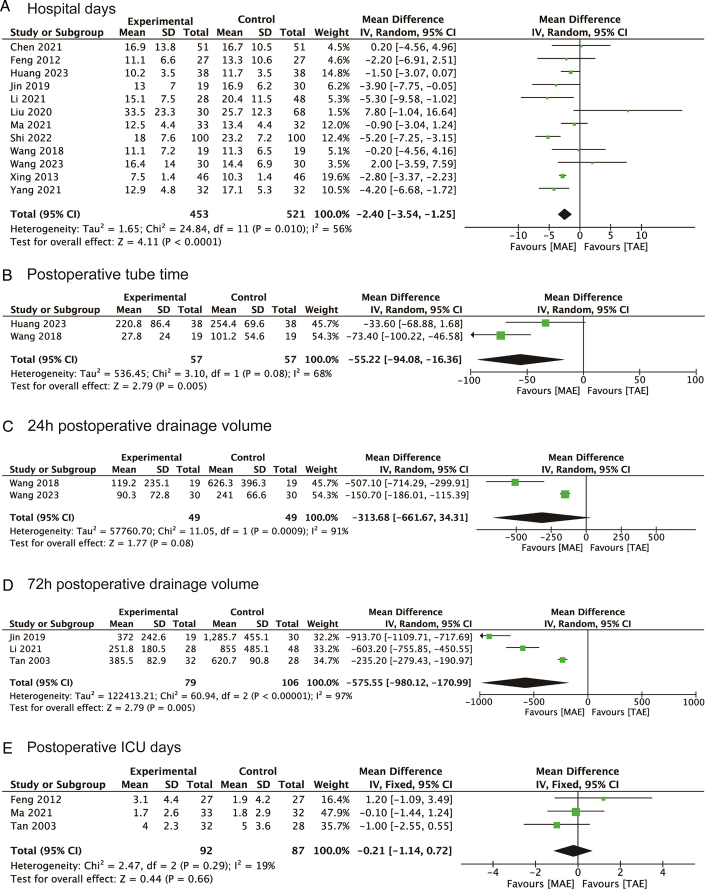
Forest plots of studies evaluating MDs of MAE versus TAE on (A) hospital days; (B) postoperative tube time; (C) 24 h postoperative drainage volume; (D) 72 h postoperative drainage volume; and (E) postoperative lCU days of esophageal cancers. MAE, mediastinoscopy-assisted esophagectomy; TAE, thoracoscope-assisted esophagectomy.

In the analysis of postoperative recovery metrics, 12 studies contributed data on the length of hospital stay following surgery. These studies uniformly indicated that patients who underwent MAE had significantly shorter postoperative hospital stays compared to those who underwent TAE, with an MD of −2.40 days (95% CI: −3.54 to −1.25), as illustrated in Figure 3A. This trend of enhanced recovery with MAE was further supported by the analysis of the total postoperative tube time. Patients in the MAE group experienced a significant reduction in tube time, with an MD of −55.22 h (95% CI: −94.08 to −16.36), detailed in Figure 3B. Additionally, the volume of postoperative drainage within the first 72 h was markedly less for the MAE group, indicating an MD of −575.55 ml (95% CI: −980.12 to −170.99), as shown in Figure 3D. This suggests a generally smoother postoperative recovery for patients in the MAE group in terms of fluid management.

However, when examining other perioperative outcomes, such as the volume of postoperative drainage within the first 24 h and the length of stay in the ICU, no significant differences were observed between the MAE and TAE groups. Specifically, the 24-h postoperative drainage volume difference was not statistically significant (MD=−313.68 ml, 95% CI: −661.67 to 34.31), presented in Figure 3C, and the postoperative ICU stay showed an MD of −0.21 days (95% CI: −1.14 to 0.72), as depicted in Figure 3E. These results highlight the specific areas where MAE provides a tangible benefit over TAE, particularly in terms of shorter hospital stays and reduced postoperative management needs, while indicating no significant advantage in certain specific perioperative outcomes.

### Postoperative complications

**Figure 4:**
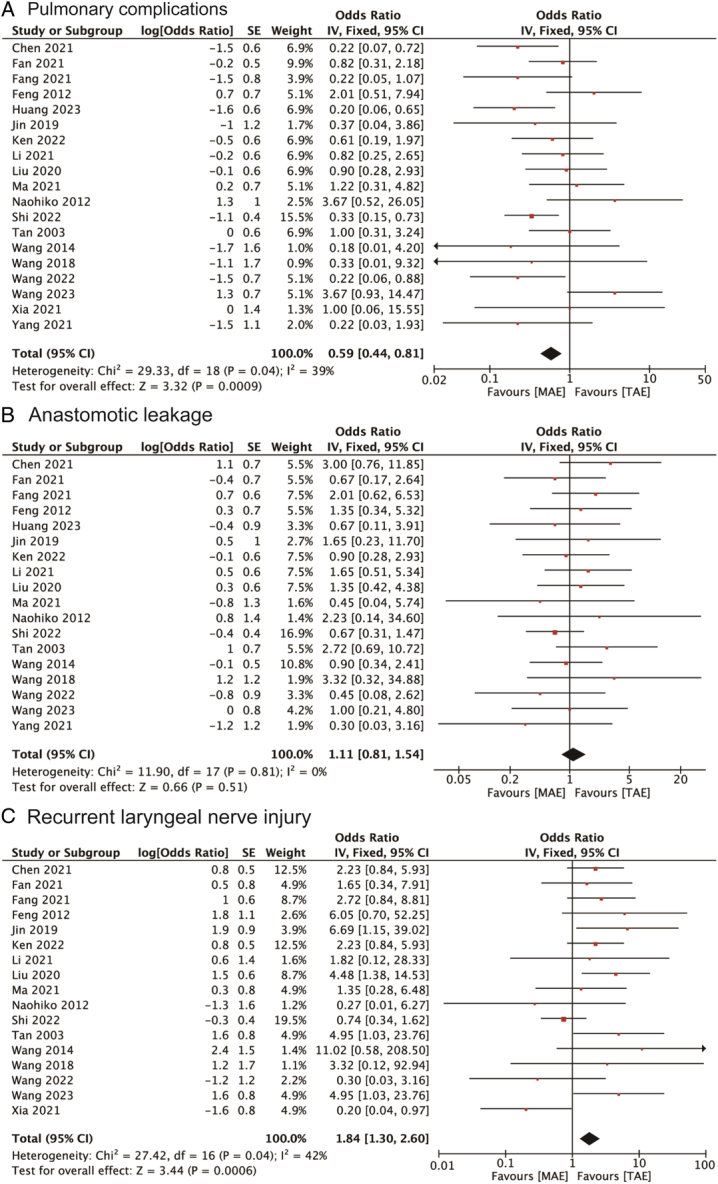
Forest plots of studies evaluating ORs of MAE versus TAE on postoperative complications of esophageal cancers, stratified by (A) pulmonary complications; (B) anastomotic leakage; and (C) recurrent laryngeal nerve injury. MAE, mediastinoscopy-assisted esophagectomy; TAE, thoracoscope-assisted esophagectomy.

Nineteen studies were reviewed to examine the relationship between MAE and the occurrence of postoperative pulmonary complications, compared with TAE. The findings highlighted a significant reduction in the incidence of pulmonary complications in the MAE group, with an OR of 0.59 (95% CI: 0.44, 0.81), as shown in Figure 4A. The analysis was conducted using a fixed-effect model due to the low heterogeneity among studies (I^2^=39%), indicating consistent results across the studies. Furthermore, the correlation between MAE and anastomotic leakage, a critical postoperative complication, was explored in 18 studies. Given the absence of heterogeneity (I^2^=0.0%), a fixed-effects model was again employed. The pooled results showed an OR of 1.11 (95% CI: 0.81, 1.54) for anastomotic leakage, indicating no statistically significant association between the surgical method and this complication, as depicted in Figure 4B. The meta-analysis also covered the incidence of recurrent laryngeal nerve injury postoperatively, based on 17 studies. The results demonstrated a higher rate of this complication in patients who underwent MAE compared to those who had TAE, with an OR of 1.84 (95% CI: 1.30, 2.60), as shown in Figure 4C.

**Figure 5:**
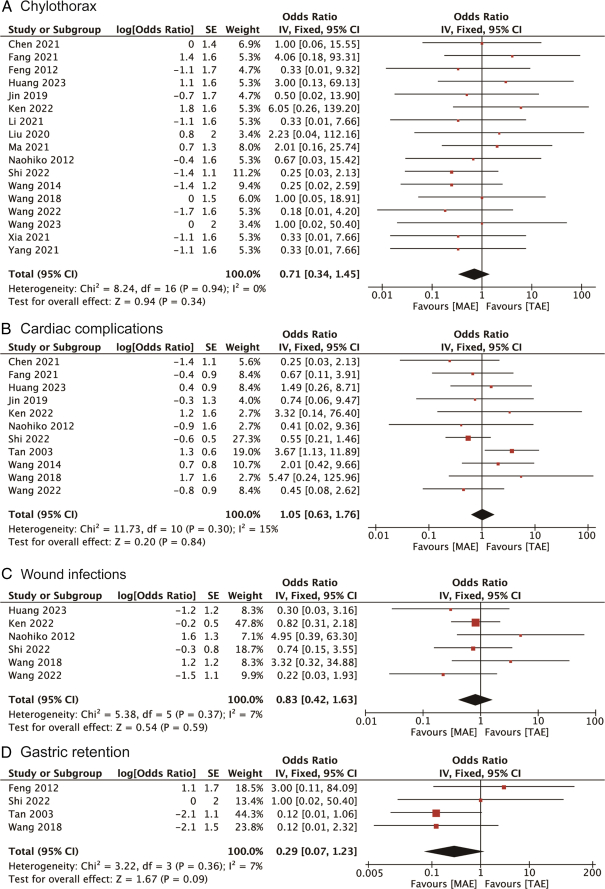
Forest plots of studies evaluating ORs of MAE versus TAE on (A) chylothorax; (B) cardiac complications; (C) wound infections; and (D) gastric retention of esophageal cancers. MAE, mediastinoscopy-assisted esophagectomy; TAE, thoracoscope-assisted esophagectomy.

Additionally, the incidences of other postoperative complications such as chylothorax, cardiac complications, wound infections, and gastric retention were investigated, comparing MAE with TAE. The ORs for these complications were 0.71 (95% CI: 0.34, 1.45) for chylothorax, 1.05 (95% CI: 0.63, 1.76) for cardiac complications, 0.83 (95% CI: 0.42, 1.63) for wound infections, and 0.29 (95% CI: 0.07, 1.23) for gastric retention, respectively, as presented in Figure 5A to D. None of these outcomes showed statistical significance, as their confidence intervals included the null value. This comprehensive analysis thus underscores the varied impacts of MAE on different postoperative complications, with a notable reduction in pulmonary complications but an increased risk of recurrent laryngeal nerve injury.

### Subgroup analyses

**Figure 6:**
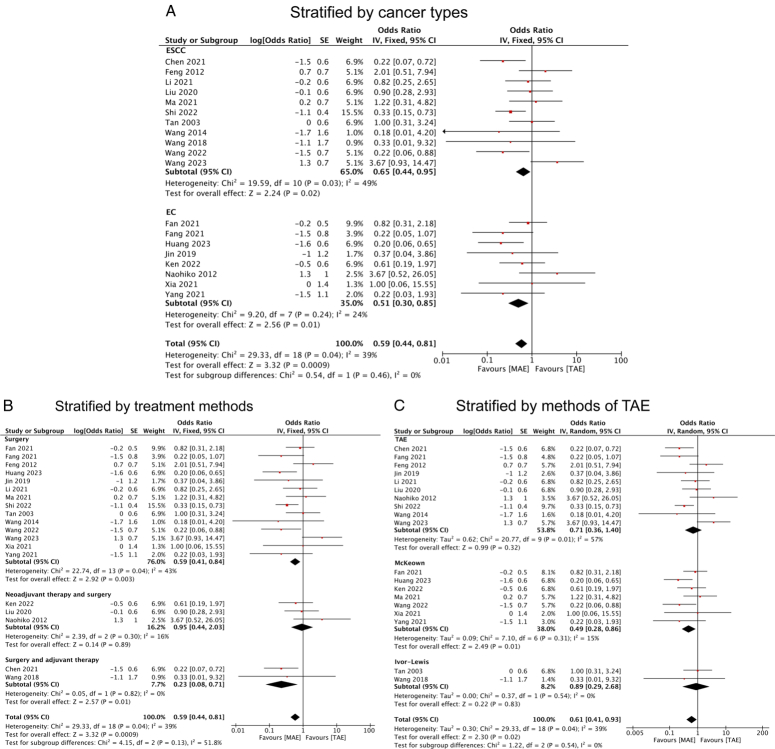
Forest plots of ORs for pulmonary complications in esophageal cancer patients who underwent MAE compared with TAE stratified by (A) cancer type; (B) treatment methods; and (C) methods of TAE. EC, esophageal cancer; ESCC, esophageal squamous cell cancer; MAE, mediastinoscopy-assisted esophagectomy; TAE, thoracoscope-assisted esophagectomy.

Given an ample volume of studies, we conducted subgroup analyses to delve deeper into the influence of MAE and TAE on these postoperative complications. Regarding postoperative pulmonary complications, stratification by different cancer types revealed a significantly lower rate in both ESCC (OR=0.65, 95% CI: 0.44, 0.95) and EC (OR=0.51, 95% CI: 0.30, 0.85) patients who underwent MAE compared to TAE (Fig. 6A). Further stratification by different treatment modalities demonstrated that patients who underwent surgery-only (OR=0.59, 95% CI: 0.41, 0.84) and surgery combined with adjuvant therapy (OR=0.23, 95% CI: 0.08, 0.71) exhibited reduced pulmonary complication rates, whereas no significant association was observed in patients who underwent neoadjuvant therapy combined with surgery (OR=0.95, 95% CI: 0.44, 2.03) (Fig. 6B). Upon detailed examination of TAE methods, MAE was identified as a protective factor against pulmonary complications compared with patients who underwent the McKeown method (OR=0.49, 95% CI: 0.28, 0.86). However, such a correlation was not observed when compared with patients who underwent the Ivor-Lewis method (OR=0.89, 95% CI: 0.29, 2.68) (Fig. 6C).

**Figure 7:**
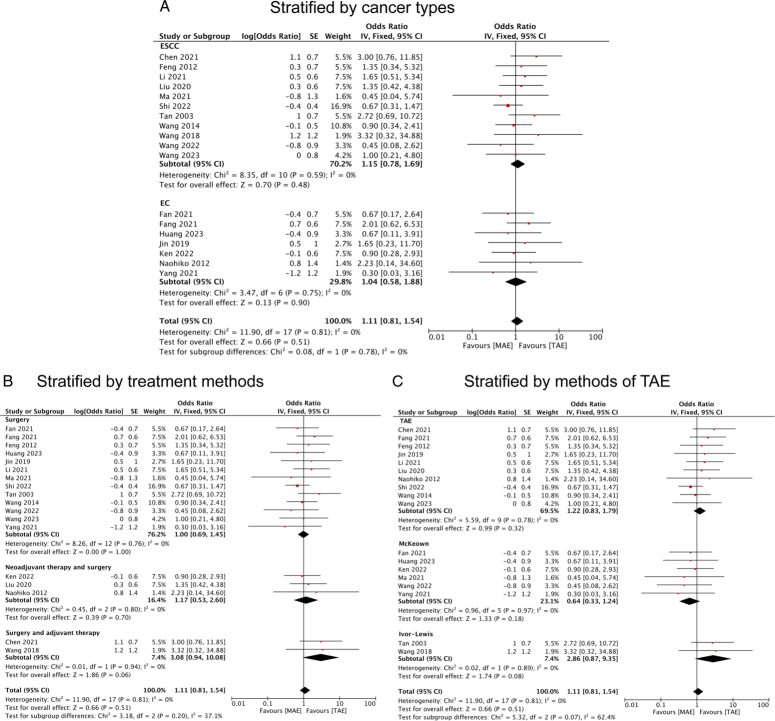
Forest plots of ORs for anastomotic leakages in esophageal cancer patients who underwent MAE compared with TAE stratified by (A) cancer type; (B) treatment methods; and (C) methods of TAE. EC, esophageal cancer; ESCC, esophageal squamous cell cancer; MAE, mediastinoscopy-assisted esophagectomy; TAE, thoracoscope-assisted esophagectomy.

Furthermore, subgroup analysis was conducted to investigate the influence of MAE on anastomotic leakage compared with TAE. However, the results indicated that no correlation was detected in postoperative anastomotic leakage, even after stratification by cancer types, treatment modalities, or TAE methods, as evidenced by their 95% CIs crossing the null line (Fig. 7A-C).

**Figure 8:**
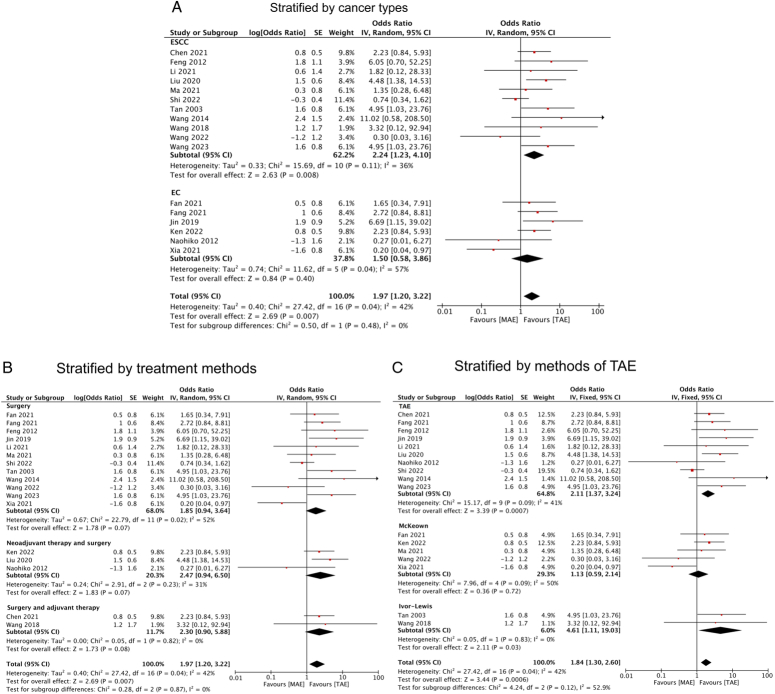
Forest plots of ORs for recurrent laryngeal nerve injury in esophageal cancer patients who underwent MAE compared with TAE stratified by (A) cancer type; (B) treatment methods; and (C) methods of TAE. EC, esophageal cancer; ESCC, esophageal squamous cell cancer; MAE, mediastinoscopy-assisted esophagectomy; TAE, thoracoscope-assisted esophagectomy.

In terms of recurrent laryngeal nerve injury, MAE was identified as a risk factor in patients with ESCC (OR=2.24, 95% CI: 1.23, 4.10) (Fig. 8A). However, upon classification by different treatment modalities, our analysis suggested that MAE might not increase the risk of recurrent laryngeal nerve injury in patients who underwent surgery-only (OR=1.85, 95% CI: 0.94, 3.64) or in combination with neoadjuvant therapy (OR=2.47, 95% CI: 0.94, 6.50) or adjuvant therapy (OR=2.30, 95% CI: 0.90, 5.88) (Fig. 8B). Furthermore, MAE was associated with a higher rate of recurrent laryngeal nerve injury when compared with patients who underwent the Ivor-Lewis method (OR=4.61, 95% CI: 1.11, 19.03). However, such an association was not observed when compared with EC patients who underwent the McKeown method (OR=1.13, 95% CI: 0.59, 2.14) (Fig. 8C).

### Postoperative survival outcomes

**Figure 9:**
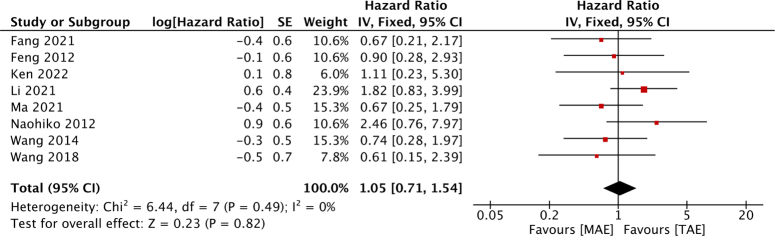
Forest plots of studies evaluating HRs of MAE versus TAE on overall survival of esophageal cancers. MAE, mediastinoscopy-assisted esophagectomy; TAE, thoracoscope-assisted esophagectomy.

The postoperative survival rate serves as a critical metric for comparing the effectiveness of different surgical methods. This outcome reflects the proportion of patients who survive for a specific period following surgery, offering invaluable insights into the long-term outcomes associated with each surgical technique. Hence, we conducted an investigation to ascertain whether MAE could impact postoperative survival outcomes compared with TAE. Among the eight studies that reported HRs of postoperative prognosis in patients with EC, the results revealed no statistical significance between these two surgical techniques (HR=1.05, 95% CI: 0.71, 1.54) (Fig. 9).

### Sensitivity analysis and publication bias

**Figure 10:**
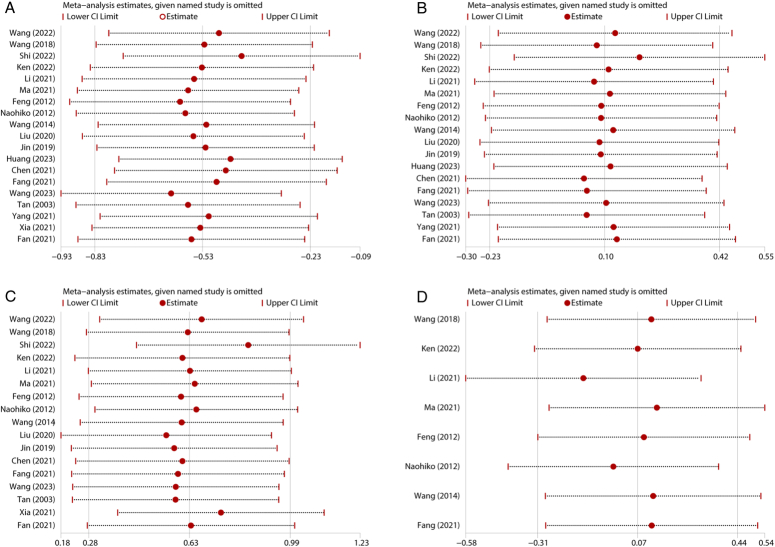
Sensitivity analysis for meta-analysis of mediastinoscopy-assisted esophagectomy compared with thoracoscope-assisted esophagectomy on (A) pulmonary complications; (B) anastomotic leakage; (C) recurrent laryngeal nerve injury; (D) overall survival.

**Figure 11:**
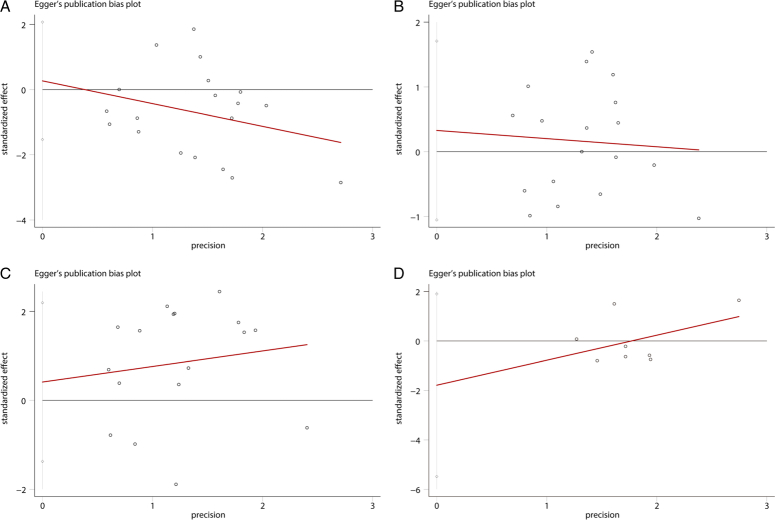
Funnel plots of publication bias for meta-analysis of mediastinoscopy-assisted esophagectomy compared with thoracoscope-assisted esophagectomy for (A) pulmonary complications; (B) anastomotic leakage; (C) recurrent laryngeal nerve injury; (D) overall survival.

The Egger’s test was applied through calculating the *P* value, and the asymmetry of the funnel plot was also utilized to detect the potential publication bias among the included studies. The results demonstrated that the *P* values of Egger’s test were 0.758, 0.621, 0.630, and 0.280 for pulmonary complication, anastomotic leakage, respectively, chylothorax, and postoperative survival outcomes (Fig. 10A-D). Thus, no significant publication bias was detected. The sensitivity analysis showed that the postoperative complications of the original analysis were not affected by removing any single study (Fig. 11A-D).

## Discussion

Currently, surgical resection is one major approach to eradicate EC. Esophagectomy is a crucial treatment option for patients with EC, particularly in cases of which the tumor is localized and has not spread extensively to other parts of the body^32,33^. With the rapid development of surgical techniques, TAE has become the preferred surgical option for EC. Nevertheless, TAE necessitates thoracic access and intricate surgical maneuvers, potentially engendering a heightened susceptibility to postoperative pulmonary complications in recovering patients. Consequently, esophageal surgeons investigated novel minimally invasive approaches of esophagectomy. In 1993, Bumm *et al.* ^7^ first reported the mediastinoscopy in EC surgery. Advancements in minimally invasive surgical techniques in the late 20th century further expanded the role of mediastinoscopy in the management of EC. This procedure circumvents the need for esophageal removal through the chest, thus minimizing the risk of lung and chest wall damage and facilitating faster postoperative recovery for patients. However, MAE might also encounter challenges such as a confined operating space and difficulty in achieving adequate local exposure, and questions have arisen regarding its potential to serve as a replacement for TAE^9^. Previous research had compared the outcomes of MAE with TAE. Due to the limited number of studies with relatively small sample sizes, the evidence still remains inconclusive^10^. Hence, we conducted this meta-analysis to comprehensively evaluate the strengths and weaknesses of MAE compared to TAE in EC surgical treatment field, aiming to provide robust evidence that can elucidate the comparative effectiveness of these surgical approaches.

Our meta-analysis stands out as the first to comprehensively integrate and analyze long-term survival outcomes. In this meta-analysis, we totally included 21 studies, of which 838 patients with EC underwent MAE and 884 patients underwent TAE. The results of this meta-analysis demonstrated that MAE could significantly reduce the operation time and decrease the blood loss during the surgery progression. The drainage in 72 h, total postoperative tube time, and postoperative hospital stay were also proved to be less in MAE group compared with patients who underwent TAE. Additionally, the postoperative pulmonary complications of MAE group were better than the TAE group. Among the postoperative complications of anastomotic leakage, cardiac complications, chylothorax, incision infection, and gastric retention were proved to have no significant differences for their pooled 95% CI crossed with the null line. The findings underscore the safety and viability of MAE as a surgical esophagectomy approach.

However, in terms of recurrent laryngeal nerve injury rate, patients who underwent MAE were shown to have a higher rate compared with TAE group. This phenomenon may arise partly due to the constrained operative field within the mediastinoscopy view during the excision of laryngeal recurrent lymph nodes, where the utilization of certain energy-based instruments or excessive force can inadvertently result in nerve injury. On the other hand, the results indicated that the number of lymph node dissection in MAE group was significantly less than that in TAE group. Due to the constrained spatial confines and visual constraints inherent to MAE procedures, the dissection of lymph nodes in the mid-mediastinum, particularly around the tracheal bifurcation, proved notably challenging. Lymphadenectomy extent is a cornerstone of EC surgery, directly influencing staging accuracy and potentially impacting adjuvant treatment decisions^34,35^. This limitation highlights the need for careful patient selection, emphasizing MAE’s suitability for early-stage EC or patients with significant operative risks that contraindicate extensive dissection. In discussing the impact of MAE on the postoperative survival rates of EC patients, our study found no statistically significant difference between MAE and TAE. It is, however, particularly worth emphasizing that mediastinoscopy presents unique advantages for use in elderly patients due to its non-requirement for thoracotomy and lower impact on pulmonary function. This aspect is especially crucial for elderly patients, who often have multiple chronic health issues, including reduced pulmonary function. On the other hand, it is important to note the limitations of MAE in patients with advanced clinical staging of lymph nodes, as mediastinoscopy may not achieve a high lymph node resection rate. In such cases, TAE might be more practical due to its efficacy in lymphadenectomy, illustrating that both surgical techniques have their respective advantages and limitations. Therefore, while the postoperative survival rate is an important metric in evaluating different surgical approaches for EC, the safety, applicability, and specific surgical outcomes, particularly regarding lymph node management, must also be considered. Mediastinoscopy offers a relatively low-risk surgical option with minimal impact on pulmonary function, making it a viable treatment strategy for elderly patients or those with compromised pulmonary function. Conversely, TAE may be preferable in cases requiring extensive lymphadenectomy. Future research should aim to refine the selection criteria for the most appropriate surgical strategy based on the specific conditions of patients, including age, pulmonary function, and lymph node staging, to improve the overall treatment outcomes and quality of life for EC patients.

Patients who underwent MAE displayed fewer postoperative pulmonary complications compared to those receiving TAE, notably in ESCC and EC cases. This benefit is linked to MAE’s avoidance of transthoracic surgery, instead requiring a minor incision beneath the neck for mediastinal access, thus reducing pulmonary risk. The advantage extends across surgery-only and adjuvant therapy cohorts, underscoring MAE’s role in minimizing surgical stress and preserving lung function. However, no significant pulmonary complication reduction was noted post-neoadjuvant therapy, suggesting potential neoadjuvant therapy effects masking MAE benefits. Indeed, neoadjuvant treatment significantly impacts surgical outcomes, which is why we have included a subgroup analysis of these patients. Our results suggested the complexity of these case, and we plan to explore this further in future studies to better understand how preoperative therapies influence surgical risks and outcomes. Comparatively, MAE outperformed the McKeown method but not significantly versus the Ivor-Lewis method, indicating different inherent surgical risk profiles.

In the subgroup analysis focusing on ESCC patients, MAE was identified as a significant risk factor for recurrent laryngeal nerve injury, with an OR of 2.24. Interestingly, when broad treatment modalities were considered—including surgery alone, neoadjuvant therapy, and adjuvant therapy—the distinction between MAE and TAE became less marked. This indicates that the method of esophagectomy might not independently predict recurrent laryngeal nerve injury but rather interacts with other factors tied to the patient’s overall treatment plan. The wide confidence intervals seen in this analysis suggest a complex interplay of factors influencing the risk of recurrent laryngeal nerve injury, potentially moderated by the disease stage, the specific technique used, or the individual patient’s anatomy. Additionally, the pronounced contrast in recurrent laryngeal nerve injury rates between MAE and the Ivor-Lewis method (OR=4.61) highlights the critical need for detailed surgical planning and precision. Conversely, the absence of a statistically significant difference between MAE and the McKeown method indicates a similar likelihood of recurrent laryngeal nerve damage, possibly due to equivalent levels of esophageal mobilization and lymph node dissection in both procedures.

Several limitations of our meta-analysis should be noticed. First, as a complex surgical procedure, the outcomes of different surgical approaches are significantly influenced by the techniques employed by individual surgeons, and the clinical heterogeneities among the studies may impact the reliability of our findings. Furthermore, given its status as a novel technology, this approach has not yet been extensively adopted, resulting in a scarcity of available studies with limited sample sizes suitable for analysis. Consequently, the diminished statistical power stemming from this constraint should be duly acknowledged. These limitations underscore the need for further research, including additional large-scale randomized controlled trials comparing MAE and TAE, to better understand the optimal application of these techniques in the treatment of EC.

## Conclusions

In our comprehensive meta-analysis comparing MAE and TAE for EC treatment, we found that MAE offers shorter operation times, less intraoperative blood loss, and reduced postoperative pulmonary complications, thus facilitating faster recovery. However, MAE also poses a higher risk of recurrent laryngeal nerve injury and results in fewer lymph nodes being dissected, which could impact its oncological efficacy in advanced disease stages. Despite these differences, long-term survival rates between the two techniques showed no significant disparity, highlighting that the choice between MAE and TAE should be based on patient-specific factors rather than inherent superiority of one method over the other. This underlines the importance of a personalized approach to surgical management in EC, emphasizing tailored treatment strategies to optimize patient outcomes. Our findings suggest that both MAE and TAE are valuable minimally invasive options, with their respective benefits and limitations warranting careful consideration in clinical decision-making.

## Ethical approval

Since this research is a review, ethical approval is not necessary.

## Consent

Since all analyses were conducted using previously published studies, patient consent is unnecessary.

## Source of funding

This work was supported by the National Natural Science Foundation of China (81970481, 82000514), Sichuan Science and Technology Program (2022YFS0048, 2021YFS0222), 1.3.5 project for disciplines of excellence, West China Hospital, Sichuan University (2020HXFH047, ZYJC18010 and 20HXJS005, 2018HXFH020), China Postdoctoral Science Foundation (2020M673241) and National Natural Science Foundation Regional Innovation and Development (U20A20394).

## Author contribution

Y.Y. conceptualized the study, revised the manuscript, and supervised the study. P.F., J.Z., and Z.L. conceptualized the study, drafted the manuscript, and made the figures. Y.L., Yu.Y., S.L., X.X., X.L., H.Z., Q.S., L.C. and X.Z. collected the data and revised the manuscript. All authors read and approved the final manuscript.

## Conflicts of interest disclosure

The authors declare no conflicts of interest.

## Research registration unique identifying number (UIN)

Registration number: CRD42024515777.

## Guarantor

Yong Yuan.

## Data availability statement

Full data are available upon request to the corresponding author.

## Provenance and peer review

Not commissioned, externally peer-reviewed.

## Supplementary Material

**Figure:**
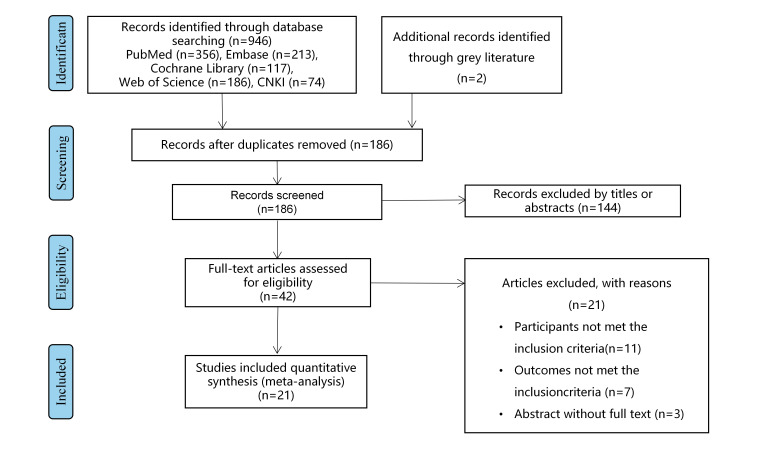


**Figure:**
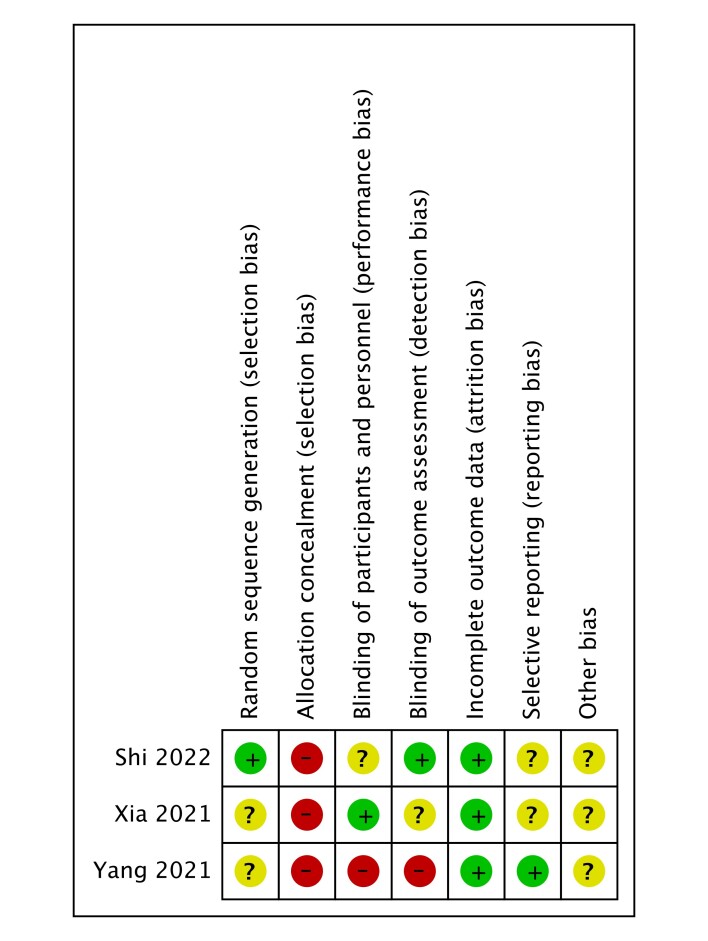


## References

[1] Sung H Ferlay J Siegel RL . Global Cancer Statistics 2020: GLOBOCAN estimates of incidence and mortality worldwide for 36 cancers in 185 countries. CA Cancer J Clin 2021;71:209–249. 33538338 10.3322/caac.21660

[2] Lagergren J Smyth E Cunningham D . Oesophageal cancer. Lancet 2017;390:2383–2396. 28648400 10.1016/S0140-6736(17)31462-9

[3] Kelly RJ . Emerging multimodality approaches to treat localized esophageal cancer. J Natl Compr Canc Netw 2019;17:1009–1014. 31390584 10.6004/jnccn.2019.7337

[4] Jin Y Lu X Xue L . Retrospective comparison of two minimally invasive esophagectomy in the treatment of esophageal cancer: pneumatic mediastinoscopy versus thoracoscopy. J Laparoendosc Adv Surg Tech A 2019;29:638–642. 30562122 10.1089/lap.2018.0512

[5] Gottlieb-Vedi E Kauppila JH Malietzis G . Long-term survival in esophageal cancer after minimally invasive compared to open esophagectomy: a systematic review and meta-analysis. Ann Surg 2019;270:1005–1017. 30817355 10.1097/SLA.0000000000003252

[6] Reichert M Schistek M Uhle F . Ivor Lewis esophagectomy patients are particularly vulnerable to respiratory impairment—a comparison to major lung resection. Sci Rep 2019;9:11856. 31413282 10.1038/s41598-019-48234-w PMC6694108

[7] Bumm R Hölscher AH Feussner H . Endodissection of the thoracic esophagus. Technique and clinical results in transhiatal esophagectomy. Ann Surg 1993;218:97–104. 8328835 10.1097/00000658-199307000-00015 PMC1242906

[8] Song S Shen C Hu Y . Application of inflatable video-assisted mediastinoscopic transhiatal esophagectomy in individualized treatment of esophageal cancer. Biomedicines 2023;11:2750. 37893123 10.3390/biomedicines11102750 PMC10603894

[9] Shi K Qian R Zhang X . Video-assisted mediastinoscopic and laparoscopic transhiatal esophagectomy for esophageal cancer. Surg Endosc 2022;36:4207–4214. 34642798 10.1007/s00464-021-08754-x

[10] Gong S Rao X Yuan Y . Clinical-pathological features and perioperative outcomes of mediastinoscopy vs. thoracoscopy esophagectomy in esophageal cancer: a meta-analysis. Front Surg 2023;10:1039615. 36865627 10.3389/fsurg.2023.1039615 PMC9971490

[11] Page MJ McKenzie JE Bossuyt PM . The PRISMA 2020 statement: an updated guideline for reporting systematic reviews. Int J Surg 2021;88:105906. 33789826 10.1016/j.ijsu.2021.105906

[12] Shea BJ Reeves BC Wells G . AMSTAR 2: a critical appraisal tool for systematic reviews that include randomised or non-randomised studies of healthcare interventions, or both. BMJ 2017;358:j4008. 28935701 10.1136/bmj.j4008 PMC5833365

[13] Yang J . Clinical application of improved inflatable mediastinoscopy in early thoracic esophageal cancer. Contemp Med 2021;27:172–173.

[14] Xia W Li X Zhang X . The effect of improved inflatable mediastinoscopy on lymph node dissection in early esophageal cancer. Inner Mongolia Med J 2021;53:579–580.

[15] Wang G Sun X Li T . Study of the short-term quality of life of patients with esophageal cancer after inflatable videoassisted mediastinoscopic transhiatal esophagectomy. Front Surg 2022;9:981576. 36684129 10.3389/fsurg.2022.981576 PMC9852052

[16] Ma J Wang W Zhang B . Minimally invasive esophagectomy via Sweet approach in combination with cervical mediastinoscopy is a valuable approach for surgical treatment of esophageal cancer. Zhong Nan Da Xue Xue Bao Yi Xue Ban 2021;46:60–68. 33678638 10.11817/j.issn.1672-7347.2021.190568 PMC10878293

[17] Wang J Wei N Lu Y . Mediastinoscopy-assisted esophagectomy for T2 middle and lower thoracic esophageal squamous cell carcinoma patients. World J Surg Oncol 2018;16:58. 29548327 10.1186/s12957-018-1361-2 PMC5857111

[18] Sasaki K Tsuruda Y Shimonosono M . A comparison of the surgical invasiveness and short-term outcomes between thoracoscopic and pneumatic mediastinoscopic esophagectomy for esophageal cancer. Surg Today 2022;52:1759–1765. 35552816 10.1007/s00595-022-02509-4

[19] Guo L Zhao Q Wang K . A case-control study on the therapeutic effect of mediastinoscope-assisted and thoracoscope-assisted esophagectomy. Surg Innov 2021;28:316–322. 32909910 10.1177/1553350620958265

[20] Feng MX Wang H Zhang Y . Minimally invasive esophagectomy for esophageal squamous cell carcinoma: a case-control study of thoracoscope versus mediastinoscope assistance. Surg Endosc 2012;26:1573–1578. 22179461 10.1007/s00464-011-2073-7

[21] Koide N Takeuchi D Suzuki A . Mediastinoscopy-assisted esophagectomy for esophageal cancer in patients with serious comorbidities. Surg Today 2012;42:127–134. 22068678 10.1007/s00595-011-0042-3

[22] Wang QY Tan LJ Feng MX . Video-assisted mediastinoscopic resection compared with video-assisted thoracoscopic surgery in patients with esophageal cancer. J Thorac Dis 2014;6:663–667. 24976988 10.3978/j.issn.2072-1439.2014.06.29 PMC4073377

[23] Nomura T Matsutani T Hagiwara N . Mediastinoscopy-assisted transhiatal esophagectomy for esophageal cancer: a single-institutional cohort study. Surg Laparosc Endosc Percutan Tech 2016;26:e153–e156. 27846176 10.1097/SLE.0000000000000348

[24] Liu W Guo X Zhao H . Mediastinoscopy-assisted transhiatal esophagectomy versus thoraco-laparoscopic esophagectomy for esophageal cancer: a single-center initial experience. J Thorac Dis 2020;12:4908–4914. 33145064 10.21037/jtd-20-1328 PMC7578494

[25] Huang ZN Liu CQ Guo MF . Clinical analysis of inflatable video-assisted mediastinoscopic transhiatal esophagectomy combined with laparoscopy. Zhonghua Wai Ke Za Zhi 2023;61:48–53. 36603884 10.3760/cma.j.cn112139-20220612-00265

[26] Chen Z Huang K Wei R . Transcervical inflatable mediastinoscopic esophagectomy versus thoracoscopic esophagectomy for local early- and intermediate-stage esophageal squamous cell carcinoma: a propensity score-matched analysis. J Surg Oncol 2022;125:839–846. 35066884 10.1002/jso.26798 PMC9304140

[27] Fang Y Chen Z Wei R . Comparison of short-term follow-up results between inflatable mediastinoscopy and video-assisted thoracoscopy combined with laparoscopic surgery for esophageal cancer. Chinese J Clin Thorac Cardiovasc Surg 2021;28:239–242.

[28] Wang Q Liu H Zhang L . Comparison of single port inflatable mediastinoscopy assisted transhiatal esophagectomy and functional esophageal cancer resection: a single center propensity score matching study. Chinese J Clin Thorac Cardiovasc Surg 2023:1–7. [online ahead of print].

[29] Tan L Xu Z qiu H . Exploring the safety of television mediastinoscopy assisted esophagectomy. Chinese J Minimally Invasive Surg 2003;05:406–407.

[30] Xing Z . Clinical analysis of television mediastinoscopy surgery for early esophageal cancer. China Pract Med 2013;8:68–69.

[31] Fan X Li H Song P . Perioperative outcomes of early esophageal cancer treated with mediastinoscopy and thoracoscopy. Chinese Electr J Chest Surg 2021;8:12–15.

[32] Luan S Xie R Yang Y . Acid-responsive aggregated gold nanoparticles for radiosensitization and synergistic chemoradiotherapy in the treatment of esophageal cancer. Small 2022;18:e2200115. 35261151 10.1002/smll.202200115

[33] Baiu I Backhus L . Esophageal cancer surgery. JAMA 2020;324:1580. 33079155 10.1001/jama.2020.2101

[34] Mimatsu K Oida T Kawasaki A . Mediastinoscopy-assisted esophagectomy is useful technique for poor surgical-risk patients with thoracic esophageal cancer. Surg Laparosc Endosc Percutan Tech 2009;19:e17–e20. 19238050 10.1097/SLE.0b013e31818aa5cc

[35] Tsurumaru M Kajiyama Y Udagawa H . Outcomes of extended lymph node dissection for squamous cell carcinoma of the thoracic esophagus. Ann Thorac Cardiovasc Surg 2001;7:325–329. 11888470

